# Issues of Acute Kidney Injury Staging and Management in Sepsis and Critical Illness: A Narrative Review

**DOI:** 10.3390/ijms18071387

**Published:** 2017-06-28

**Authors:** Christian Nusshag, Markus A. Weigand, Martin Zeier, Christian Morath, Thorsten Brenner

**Affiliations:** 1 Department of Nephrology, Heidelberg University Hospital, 162, Im Neuenheimer Feld, D-69120 Heidelberg, Germany; christian.nusshag@med.uni-heidelberg.de (C.N.); Martin.Zeier@med.uni-heidelberg.de (M.Z.); Christian.Morath@med.uni-heidelberg.de (C.M.); 2 Department of Anesthesiology, Heidelberg University Hospital, 110, Im Neuenheimer Feld, D-69120 Heidelberg, Germany; Markus.Weigand@med.uni-heidelberg.de

**Keywords:** acute kidney injury, biomarkers, disease staging, glomerular filtration rate, renal replacement therapy, timing

## Abstract

Acute kidney injury (AKI) has a high incidence on intensive care units around the world and is a major complication in critically ill patients suffering from sepsis or septic shock. The short- and long-term complications are thereby devastating and impair the quality of life. Especially in terms of AKI staging, the determination of kidney function and the timing of dialytic AKI management outside of life-threatening indications are ongoing matters of debate. Despite several studies, a major problem remains in distinguishing between beneficial and unnecessary “early” or even harmful renal replacement therapy (RRT). The latter might prolong disease course and renal recovery. AKI scores, however, provide an insufficient outcome-predicting ability and the related estimation of kidney function via serum creatinine or blood urea nitrogen (BUN)/urea is not reliable in AKI and critical illness. Kidney independent alterations of creatinine- and BUN/urea-levels further complicate the situation. This review critically assesses the current AKI staging, issues and pitfalls of the determination of kidney function and RRT timing, as well as the potential harm reflected by unnecessary RRT. A better understanding is mandatory to improve future study designs and avoid unnecessary RRT for higher patient safety and lower health care costs.

## 1. Introduction

Acute kidney injury (AKI) is a disease entity with a high incidence on intensive care units (ICU) [1,2]. Up to 57% of all critically ill patients suffer from AKI [3] and in 50% of these cases, sepsis appears to be the most frequent contributing factor [1]. Mechanistically, the pathophysiology of AKI in sepsis has to be kept apart from ischemic AKI, where systemic hypotension and reduced renal blood flow (RBF) result in acute tubular necrosis. In septic AKI, strong evidence supports an RBF-independent microcirculatory dysfunction in renal parenchyma induced by inflammatory mediators, immune cell infiltration, and the deregulation of nitric oxide synthase [4,5]. The resulting redistribution of blood flow from renal medulla to the cortex, as well as a general deterioration of microcirculatory oxygenation, lead to tubular stress/injury and the formation of reactive oxygen species [4]. However, low blood pressure and renal hypo-perfusion also contribute to renal injury in septic shock.

In general, septic AKI has to be considered as a syndrome [5], characterized by the fulfillment of both the new Sepsis-3 criteria [6] and the AKI definition by Kidney Disease Improving Global Outcomes (KDIGO) [7]. The total AKI-associated mortality is thereby high (40–60%) [1,2,8,9] and the corresponding short- and long-term outcomes in the form of chronic (CKD) or end-stage renal disease (ESRD) are devastating [9,10]. The incidence of RRT-requiring AKI thereby accounts for 4–8% [1,2,11] and about 12–25% of these patients remain RRT-dependent [1,8,9]. In total, this represents a heavy burden on health care providers, where RRT is one of the most driving cost factors [12,13]. In addition, the quality of life of ICU patients treated with RRT is essentially impaired compared to other long-term critically ill survivors [14,15,16]. In order to improve AKI therapy and (long-term) outcomes in sepsis and critical illness, the precise measurement of renal function, reliable AKI staging, and optimal timing of renal replacement therapy (RRT) are essential. A better understanding is needed in light of the potential harm of RRT procedures and substantial health care costs. This review critically assesses our current knowledge of AKI staging, reviews problems in the determination of kidney function, and discusses the difficulties surrounding the timely initiation of RRT in order to give rise to potential improvements in AKI management and future study designs.

## 2. Staging of Acute Kidney Injury

The most recent concept of AKI staging was published by the Kidney Disease Improving Global Outcomes (KDIGO) group in 2012 [7] (Table 1) and defines AKI by changes in serum creatinine (SCr) and/or urine output (UO).

KDIGO merged the Acute Kidney Network (AKIN)- and RIFLE (Risk, Injury, Failure, Loss, End-Stage Kidney Disease)-criteria [17,18] to improve the sensitivity of AKI diagnosis and assessment of related in-hospital mortality [2,7,19]. This was achieved by merging both the absolute SCr rise from the baseline (AKIN: SCr ≥ 0.3 mg/dL or SCr ≥ 1.5-fold, RIFLE: only SCr ≥ 1.5-fold) and the time periods of SCr increases used by RIFLE and AKIN (AKIN: <48 h, RIFLE: within 1–7 days and >24 h). It is noteworthy that only slightly modified SCr and UO criteria are likewise integrated in the new sepsis definition via the Sepsis-related Organ Failure Assessment (SOFA) Score [20]. The SOFA score provides the basis for the international consensus definition of sepsis and septic shock [6] and emphasizes the importance of sepsis-related organ failure in critical illness.

The definition of baseline SCr, however, affects AKI classification. KDIGO suggest the lowest SCr within the first seven days of admission. The way to define baseline SCr is controversial [21,22,23,24,25,26]. A recent study found that the mean of the outpatient SCr values from seven to 356 days before admission was the most reliable number [21]. However, the most recent outpatient SCr was only slightly inferior and is much more applicable in clinical practice [21]. But often preexisting blood results are often absent and only the lowest inpatient SCr gives an idea about the baseline kidney function. The occasionally suggested back-calculation of baseline SCr by reversing the Modification of Diet in Renal Disease (MDRD) equation—assuming a normal glomerular filtration rate (GFR) of 75 mL/min—needs to be used with caution, especially in elderly patients [25,26]. Due to unknown preexisting CKD, the calculated baseline SCr may be underestimated [23]. Without having any information about the former kidney function and patient’s history, sometimes only a normal SCr value after a passed AKI episode proves the initial degree of AKI severity. It is noteworthy that if SCr does not return to normal levels, a differentiation between a pre-existing or a new onset CKD is challenging. The determination of parathormone levels and ultrasound scans of the kidneys are crucial to answer this question [27,28]. Thereby, elevated parathormone levels, as a marker for an impaired calcium-phosphate metabolism, and kidneys small in size, are major signs for chronic renal damage [27,28]. Nevertheless, the definition of baseline SCr can significantly influence AKI staging.

### Outcomes and Staging

Recent studies investigated the ability of the KDIGO criteria to predict the outcome of AKI [29,30]. Compared to earlier studies [31,32,33], the authors showed an increased in-hospital mortality rate with a higher AKI stage, with odds ratios (OR) of 1.68 (95% Confidence Interval (CI) 0.74–3.84), 2.25 (95% CI 0.91–5.56), and 9.40 (95% CI 4.26–20.70) for AKI stage I, stage II, and stage III, respectively. Patients who fulfill both the SCr and UO criteria exhibit the highest long- and short-term mortality- and RRT-rates [29,30,34,35]. The KDIGO classification thereby prevails with a higher sensitivity for AKI diagnosis and is more predictive for hospital mortality compared to RIFLE and equivalent to AKIN [19]. A more detailed analysis of all 10 KDIGO-AKI definitions (Table 1) recently showed a remarkable difference in hospital mortality [29]. The UO criteria were linked to the highest mortality rate in any stage, with a 20% higher mortality rate in stage 3 compared to the corresponding SCr-based definition. The mortality was only higher in patients receiving RRT. Taken together, the mortality rate in stage 3 with its five definitions (Table 1) ranged from 27.6 to 55.6%, with SCr > 4 mg/dL representing the lowest mortality rate (27.6%). These findings emphasize a huge outcome variability within AKI stages, most notably in stage 3. A study by Vaara et al. analyzed the association between UO and the subsequent development of AKI by SCr-criteria, the need for RRT, and long-term mortality [35]. UO from <0.1 mL/kg/h for 3–6, 6–12 and 12–24 h were significantly associated with an increased risk of AKI, defined by SCr or the need for RRT with an OR of 2.17 (95% CI 1.35–3.48), 2.95 (95% CI 1.20–7.25), and 5.74 (95% CI 1.67–19.73), respectively. Comparable results were found for 90-days mortality. An UO of 0.1 to <0.3 mL/kg/h for more than 6 h and periods of UO < 0.1 mL/kg/h for longer than 3, 3–6, 6–12, 12–24, and >24 h were related to an increase in 90-days mortality with an OR of 1.96 (95% CI 1.13–3.38), 2.08 (95% CI 1.27–3.42), 3.04 (95% CI 1.50–6.15), 6.78 (95% CI 2.79–16.44), and 9.76 (95% CI 4.07–23.43), respectively. This emphasizes that the reduction in UO represents an independent risk factor for increased 90-day mortality.

Hence, these results may challenge the AKI staging by KDIGO due to a huge variation in the outcome parameters between SCr- and UO-criteria of the same AKI stage, most notably within the UO range <0.3 mL/kg/h. In the future integration of “renal damage” biomarkers (Table 2) in AKI, scoring may improve early AKI diagnosis, the staging and outcome predicting ability of AKI-staging, and might help to predict the potential need for RRT in the further clinical course [36,37,38].

## 3. Estimated Glomerular Filtration Rate and Its Limitations on Intensive Care Units

Ideally, and certainly in an intensive care unit (ICU) setting, knowledge of the real time GFR should not only provide the exact level of kidney dysfunction, but should also allow a more accurate dosing of drugs. Especially in sepsis patients, an adequate dosing of antibiotics is of great relevance [45].

Unfortunately, the SCr derived from the estimated glomerular filtration rate (eGFR) has major limitations in critically ill patients. After a single renal insult—for instance a period of ischemia—it takes at least 24–72 h before a new steady state in SCr levels is established [46,47]. Consequently, it is misleading to rely on actual SCr values for calculating the GFR. Multiple insults and variable SCr kinetics, as well as changes in the volume of distribution (VOD) and alterations in creatinine generation, further complicate the definition of renal function [48,49]. The “Chronic Kidney Disease Epidemiology Collaboration” (CKD-EPI)- and “Modification of Diet in Renal Disease” (MDRD)-formulas to calculate eGFR have been developed in stabile CKD [50,51] and are thus inappropriate for use in AKI [52]. A recent study evaluated the performance of measured creatinine clearance (CrCl) and GFR estimations by MDRD, CKD-EPI, and Cockcroft-Gold in comparison to a gold standard GFR measurement by chromium-ethylenediaminetetraacetic acid (^51^Cr-EDTA) [49]. As expected, CrCl showed poor precision due to the non-existing steady state during urine collection, but GFR equations showed an even higher error when applied. Thus, CrCl with short urine collection periods (2–4 h) might currently be the most feasible and reliable GFR measurement method for ICU patients [53]. This time frame reduces the likelihood for errors caused by non-steady state conditions, inaccurate time-recording, and incomplete urine collection [53,54,55,56]. However, CrCl still tends to overestimate the GFR by 10–20% due to GFR independent tubular creatinine secretion [57].

A newer, functional, endogenous GFR marker is Cystatin C. Cystatin is a 13 kD cysteine proteinase inhibitor [58] produced at a constant rate by almost every nucleated cell and exclusively eliminated via glomerular filtration [59,60]. In contrast to SCr, it is less influenced by muscle mass, age, sex, and race, and has one third of the VOD of SCr, allowing a faster steady state attainment and earlier AKI detection [60,61,62,63]. However, the superiority of Cystatin C over SCr for GFR estimation in critical illness [64,65,66,67], as well as the impact of inflammation and sepsis on cystatin C levels, is doubtful [68,69,70,71,72,73]. Furthermore, glucocorticoid therapy, thyroid disease, cancer, smoking, obesity, and diabetes may all substantially affect the Cystatin C serum concentration [68,69]. Thus, more reliable functional biomarkers with a small VOD and less metabolic interference are needed. The gold standard methods for GFR estimation, which repose on the clearance of exogenous marker (^51^Cr-EDTA, inulin), are not feasible on ICUs due to high costs and cumbersome assays [52].

## 4. Renal Replacement Therapy—Pitfalls and Timing

**Figure 1:**
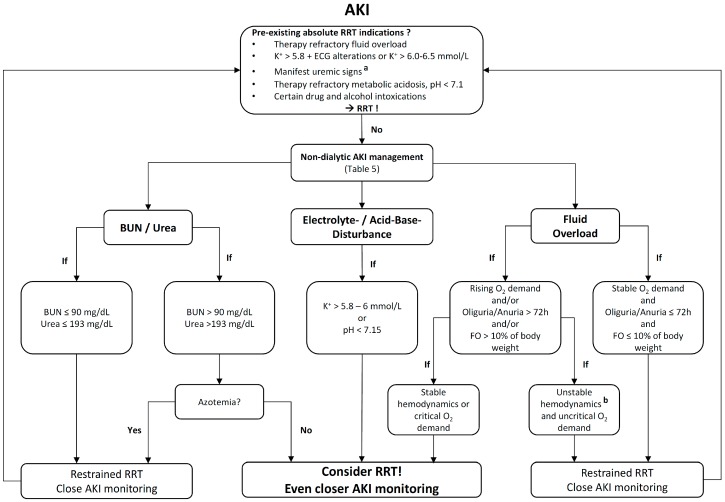
Algorithm for the initiation of renal replacement therapy (“Heidelberg Standard”) [74,75]. AKI, Acute kidney injury; BUN, Blood urea nitrogen; FO, Fluid overload; FiO_2_, Fractional inspired oxygen; K^+^, Potassium; O_2_, Oxygen; RRT, Renal replacement therapy. ^a^ Unexplained decline in mental status, nausea, vomiting, bleedings due to thrombocytophatia, pericarditis, pruritus. ^b^ RRT might be beneficial despite unstable hemodynamics, especially in cardiac decompensation or critical O_2_ demand. An individual risk-benefit analysis is mandatory.

There are clear indications for the initiation of RRT (Figure 1). The optimal timing in the absence of these classic indications, however, is an ongoing matter of debate. Decreasing kidney function, electrolyte-/acid-base-disturbance, aggravating fluid overload (FO), and the progressive involvement of extra-renal organs are still the main decision criteria. The applied clinical and laboratory thresholds for RRT-initiation are controversial. In addition, confounding factors, which influence the blood urea nitrogen (BUN)/urea and SCr concentration, apart from kidney function, are often missed and may lead to a misjudgment in the diagnosis and severity of AKI, as well as RRT management.

### 4.1. Role of Serum Creatinine

Creatinine is an alkaline amid that is derived from the metabolism of skeletal muscle and from dietary meat intake. Mechanistically, one has to consider three distinct factors that influence the SCr concentration independently from kidney function: variation in creatinine production, tubular secretion, and analytical measurement issues.

### 4.2. Variation in Creatinine Production

The mean SCr values for men and women are 1.16 (median 1.09, 95th percentile 1.40) and 0.96 mg/dL (median 0.89, 95th percentile 1.18), respectively, and are higher in people of an Afro-American ethnicity, with values of 1.25 mg/dL in men (mean 1.16, 95th percentile 1.53) and 1.01 in women (median 0.92, 95th percentile 1.23) [76]. These differences are primarily explained by changes in body composition. Hence, old age, amputations, muscle wasting conditions, and malnutrition are associated with reduced creatinine production [77]. Especially in critical illness, malnutrition, an insufficient protein supply, and immobilization, the result of catabolism and muscle wasting can cause a significant drop in SCr levels [49,78,79]. Likewise, liver cirrhosis diminishes creatinine formation, secondary to decreased hepatic creatine synthesis [80], and reliable evidence supports an independent reduction in creatinine generation under septic conditions [81]. Considering that almost 50% of AKI cases on IUCs are sepsis-related [1,4], this is a relevant detail to keep in mind. In addition, an increase in the total body water level can mimic potential renal recovery or attenuates AKI severity by SCr dilution [48,82,83,84].

Other factors can lead to an artificial SCr increase such as a disproportional rise in SCr due to rhabdomyolysis. According to different authors, values beyond 2.0 up to 2.5 mg/dL/24 h or a disproportional increase in SCr compared to urea can indicate ongoing rhabdomyolysis [85,86,87]. In contrast, when the kidney function is totally lost, it is generally believed that SCr rises by approximately 1.0–1.5 mg/dL/24 h and does not exceed 2.0 mg/dL per day [85].

### 4.3. Variations in Tubular Creatinine Secretion

In total, proximal tubular creatinine secretion accounts for 10–20% of the total CrCl and increases to 50% in CKD when the GFR is dropping. Thus, CKD patients with a true GFR of 60–80 mL/min may still have an SCr value below 1 mg/dL [88,89,90]. Once the SCr level exceeds 1.5–2 mg/dL, the tubular secretory capacity is saturated and further injury leads to the expected rise in SCr [91].

More relevantly for ICU patients is drug interference with these organic cation secretory pathways. The drugs diminish proximal tubular creatinine secretion and lead to a GFR independent SCr rise, without an awareness of a misjudgment of kidney function. The SCr rise is reversible and normally does not exceed a range from 0.4–0.5 mg/dL, but might be higher in CKD patients when creatinine secretion is upregulated. Drugs that interfere with such pathways are trimethoprim, dronedarone, ranitidine, and cimetidine [49,92,93,94,95].

### 4.4. Creatinine Measurements

Drugs additionally interact with creatinine assays. The alkaline picrate method (Jaffé assay)—the standard colourimetric assay to determine SCr concentrations—typically interferes with other chromogens like cefoxitin and flucytosine [96,97]. This results in an artificial, kidney-independent increase in SCr. Clinically relevant is the interference of the Jaffé assay and acetoacetate in diabetic ketoacidosis. It can lead to rises of SCr from 0.5 to >2 mg/dL or more [98,99,100,101]. Other compounds that may artificially increase the colourimetric SCr concentration are glucose, IgM paraproteins, ketoacids, uric acid, hemolysed hemoglobin >600 mg/dL, ascorbic acid, cephalosporines, and dopamine [102,103]. The more expensive enzymatic assays are considered to be more precise, with less interference [102,104]. In contrast, hyperbilirubinemia affects the assays by decreasing the SCr levels. This effect has progressive dynamics beyond serum bilirubin concentrations of 9.9 and 19.9 mg/dL for kinetic Jaffé and enzymatic assays, respectively [102,104]. The magnitude of bilirubin interference is laboratory dependent, ranging from 1.9 to 30 mg/dL [80].

Even today, a universal and standardized creatinine assay is missing. SCr alterations ± 0.3 mg/dL between different laboratories are common [57]. In a study evaluating over 5000 laboratories, the overall mean SCr value ranged from 0.84 to 1.21 mg/dL when measuring a standardized sample by Jaffé or an enzymatic assay [99]. All factors which influence SCr levels independent from kidney function are summarized in Table 3.

### 4.5. Role of Blood Urea Nitrogen/Urea

Blood urea nitrogen (BUN) is a derived measure for the blood nitrogen content of urea (BUN mg/dL × 2.142 = urea mg/dL). With an average protein diet, a stable volume status, and balanced electrolytes, the ratio of BUN to SCr (BUN/SCr) in healthy individuals is approximately 10:1 [107,108]. Nitrogen retention occurs when the GFR is reduced to less than 30%. However, in contrast to SCr, the rate of urea production is less constant and altered by several factors beyond renal function [108,109].

Under normal conditions, 40–50% of the filtered urea is passively reabsorbed in the tubule to preserve a sufficient osmotic gradient in the renal medulla. In cases of volume depletion, urea reabsorption increases passively as a function of increased proximal sodium and water reabsorption, and further by antidiuretic hormone mediated diffusion in the collecting duct [108,110]. Altogether, this leads to a rise in BUN out of proportion to GFR and SCr.

The amount of BUN/urea formation depends on four aspects: dietary protein supply, rate of amino acid incorporation into tissue (anabolism), amino acid release from tissue (catabolism), and liver capacity to form urea [108]. Any variation in these metabolic pathways results in a disproportional BUN/SCr dissociation. Thus, a low protein diet or liver disease might be associated with near-normal BUN levels, despite a reduction in GFR [80,111]. In contrast, a high-protein diet, intestinal blood loss, and accelerated tissue breakdown due to malnutrition, trauma, glucocorticoid therapy, and immobilization are causes of an elevated urea production [79,108,112,113]. This primarily kidney-independent rise in blood nitrogen metabolites is called azotemia and should be considered apart from situations where urea serves as the surrogate parameter for other retained metabolites due to renal impairment (“uremia”). In accordance with data where “uremic symptoms” occur after urea loading when the urea levels exceed 300 mg/dL [114], a more delayed RRT initiation might be justified in azotemia. The identification and remediation of the underlying trigger factor is crucial in this context. Apart from azotemia, however, the latest data by Gaudry et al. supports a restrictive RRT initiation as long as the BUN level remains below 90 mg/dL (~urea 193 mg/dL) [74]. The safety of a delayed RRT initiation above this BUN level remains unclear.

### 4.6. Timing of Renal Replacement Therapy

The definition of “early” versus “late” RRT Initiation is controversial. Consequently, the related thresholds of BUN/urea, SCr [115,116], and UO [117] vary enormously across all studies and make them difficult to compare [118]. The study quality is thereby extremely heterogenic and the disease severity differs substantially [115,117,119].

Table 4 summarizes all relevant studies according to their study design and gives an overview of the variability of applied “early” and “late” RRT criteria, the final pre-dialytic SCr, and BUN/urea study cutoffs, as well as the corresponding mortality. Studies prior to 1990 are excluded in agreement with other authors due to improved RRT expertise and intensive care management [119]. In this context, meta-analyses [115,117,119], and especially observational studies [120,121,122,123,124], recently showed a tendency towards a reduced mortality or improved renal recovery under “early” RRT. This result is crucially driven by observational studies with significant heterogeneity and study quality [115,119]. However, after exclusion of observational studies, meta-analyses, as well as two recent RCTs showed no mortality differences [74,125].

In the end, a general problem across all studies that remains is the distinction between beneficial and unnecessary “early” RRT due to spontaneous renal recovery. Recent data supports this issue by showing a higher autonomous renal recovery rate in the “late” group and a delayed recovery under “early” RRT [74,116,125]. Gaudry et al. even showed an autonomous renal recovery of 49% in the “late” RRT group, with the lowest associated mortality (37.1%). However, patients in the “late” group without renal recovery revealed the highest mortality (61.8%). This might indicate that the early identification of patients without autonomous recovery, rather than RRT timing, is the real point of interest. Nevertheless, in the single center study by Zarbock et al., the mortality was significantly lower under “early” RRT [126]. However, the result may have been influenced by a diminished timeframe for autonomous renal recovery due to an RRT that was applied relatively early in the “late” RRT group in relation to other studies (Table 4) [74,126]. Hence, current data remains conflicting.

Taken together, RRT initiation before the development of life-threatening complications is desirable. The use of clear “cutoffs” (crea, urea/BUN) for RRT initiation remains questionable and is not recommended by the KDIGO guidelines. In rising FO, RRT should be applied restrictively as long as oliguria persists for less than 72 h, FO accounts for less than a 10% increase of body weight, and the oxygen demand remains stable [74,75]. In more severe cases of FO, RRT initiation remains an individual, clinical decision dependent on the underlying illness, hemodynamic stability, volume dependent cardiac output, and oxygen demand. Keeping these issues in mind, Figure 1 (“Heidelberg Standard”) provides an algorithm for RRT initiation.

In future study designs, the use of biomarkers for RRT timing and the identification of high risk patients might be of great interest. However, a better understanding of their role in heterogeneous AKI pathophysiology and the establishment of biomarker thresholds, which sufficiently predict AKI and outcomes in heterogeneous cohorts, are indispensable.

In this regard, the cell cycle arrest biomarkers “tissue inhibitor of metalloproteinase-2” (TIMP-2) and “insulin like growth factor binding protein-7 (IGFBP-7), which can be measured with a point-of-care device (Nephrocheck) [47], may represent the first milestones in this field. They have already proven a high diagnostic potential in predicting moderate and severe AKI 12 h after admission [41,42,127], but need to be validated for RRT initiation. As G_1_-cell cycle arrest molecules, both biomarker possess the advantage of indicating a very early impairment of (tubular) renal function and are verifiably involved in AKI pathogenesis [39,128,129]. In the future, not one, but rather a cluster of biomarkers (Table 2) may be needed to sub-classify heterogeneous AKI pathophysiology in order to improve the prediction of AKI with a need for RRT, as well as AKI diagnosis, staging, and long-term outcomes [130].

## 5. Potential Harm of Renal Replacement Therapy

Known from RRT initiation in ESRD [147], the subsequent loss of residual kidney function might also occur in AKI patients. In addition, renal recovery appears to be delayed after the “early” initiation of RRT [74,116,125]. The underlying factors which negatively affect renal function and recovery thereby rely on three mechanisms: decline in UO, RRT induced hypotension, and interference with inflammatory pathways. Thus, an optimal RRT timing and non-dialytic AKI management, which buys time for autonomous renal recovery, are crucial to reduce unnecessary RRT (Table 5).

### 5.1. Decline in Urine Output

RRT initiation is frequently accompanied by a fall in UO as a function of volume and urea removal [147,158]. However, so far, there is no reliable evidence that reduced UO by itself compromises renal recovery. Most patients who require RRT have less than 5–10% of their nephrons functioning and alterations in UO only take place in these remaining nephrons [159]. Consequently, reduced UO does not delay or alter the regeneration of the 90–95% of nephrons that are no longer functioning. In accordance, increasing UO via loop diuretics neither shortens AKI duration nor reduces the need for RRT, and might be associated with less renal recovery and an increased risk of death [154,160,161,162,163,164,165]. However, an appropriate use of diuretics is justified to prevent FO and electrolyte imbalance, particularly to facilitate sufficient mechanical ventilation [75,161,162,166].

### 5.2. Renal Replacement Therapy-Induced Hypotension

Decreased renal perfusion due to intravascular volume depletion, decreased cardiac output, and systemic hypotension are the most common mechanisms which prolong the course of renal injury [167,168]. The situation is aggravated by an impairment of renal autoregulation in AKI that leads to a microvascular imbalance, even with small changes in the systemic blood pressure [169]. Consequently, ischemia and reperfusion lead to the impairment of tubular transport mechanisms and acute tubular necrosis [4,170,171]. Especially in septic AKI, the drop in the tubular reabsorptive capacity might result in high sodium and chloride concentrations in distal tubules, which is then sensed by the macula densa and leads to a drop in the GFR via negative tubular glomerular feedback [171,172]. Furthermore, there is strong evidence that ischemic AKI impairs the expression of organic anion transporters (OAT)—essential for the excretion of toxic compounds—in proximal tubular cells. This mechanism is mediated by metabolites of cyclooxygenase 1 (COX-1), especially prostaglandin E2 [173]. The consecutive accumulation of toxic compounds might explain the resulting negative effects on renal perfusion, post-ischemic histological alterations, increased apoptosis, and on renal outcome, determined by GFR. In this context, Indomethacin, a COX-1 inhibitor, showed a remarkable ability to restore sufficient OAT expression and protection against renal damage after ischemic AKI, without affecting renal perfusion [174].

Returning to the underlying mechanism of RRT-induced hypotension, fluid removal and the derangement of solutes remain the primary causes [175]. Thus, hemofiltration procedures with less solute interference and a continuous type of application with gentle fluid removal, theoretically permit a higher hemodynamic stability than hemodialysis [176,177,178,179]. Despite these theoretical advantages of hemofiltration, the data on this issue is inconclusive. However, the hemodynamic instability during dialysis is not that relevant in clinical practice, especially in the Age of hybrid procedures like sustained low efficiency dialysis (SLED) [180,181,182,183]. In this context, observational studies have shown a tendency towards improved renal recovery under continuous hemofiltration, whereas a recent meta-analysis (excluding observational studies) and RCTs showed no difference [184,185,186,187]. The corresponding mortality rates were comparable in these studies. Hence, in accordance with recent data, there is no reliable evidence justifying a general preference for hemofiltration over dialysis procedures in clinical practice [181,188,189].

### 5.3. Activation of Inflammatory Pathways

Renal recovery may be hampered by the activation of inflammatory pathways via blood-dialyzer interaction [190]. At least in part, this is mediated by the upregulation of adhesion molecules like “intracellular adhesion molecule-1” (ICAM-1) on granulocytes. The level of complement activation is dialyzer membrane-dependent. Particularly, synthetic “biocompatible” membranes (polysulfone, polycarbonate, polymethylmethacrylate = PMMA)—in contrast to cuprophane—activate complement to a lesser extent and cause less neutropenia [191,192]. In most, but not all clinical studies, the use of biocompatible membranes was associated with higher renal recovery rates and improved survival [193,194,195,196]. Another relevant aspect during RRT might be the clearance of inflammation mediators, especially in septic AKI. On one hand there is evidence that pro-inflammatory cytokines like interleukin (IL) 6, IL 8, IL 10, and TNFα are associated with increased mortality in septic AKI and might be blamed for septic organ failure [4,197,198,199,200,201]. Thus, the unselective clearance of pro-inflammatory cytokines, mostly by adhesion to PMMA membranes or others [202,203,204], might promise beneficial effects on outcomes in sepsis [205]. On the other hand, cytokine clearances of each cytokine vary enormously and depend on the applied RRT procedures, as well as the filter membrane used [206]. Hence, the clearance of beneficial anti-inflammatory cytokines like IL-22 through RRT could also be a potential cause of harm for renal recovery [207,208]. Prospective, randomized clinical trials with “high cut off” membranes are needed to develop a better understanding of cytokine clearance and its effect on clinical outcomes in sepsis and heterogeneous AKI pathophysiology [209,210,211].

## 6. Conclusions

AKI is a major complication in critically ill patients suffering from sepsis or septic shock. Even moderate rises in SCr correlate with adverse short- and long-term outcomes. Thus, a differentiated AKI staging, which predicts future clinical outcomes, is mandatory for an appropriate initiation of therapeutic measures. Thereby, an independent clinical evaluation of crea und UO criteria in AKI staging—due to enormous outcome variation—is indispensable. Furthermore, the knowledge of the “true” GFR is fundamental in septic and critically ill patients. The at present methods used for estimating renal function are neither feasible in an ICU setting nor give an accurate estimate of “true” GFR. The establishment of a more reliable, functional marker for GFR-measurement is needed. The ability of BUN/urea and SCr to serve as an independent parameter for RRT initiation in AKI is questionable. However, taking clinical observations, potential confounders, and disease-related and dietary aspects into account, a differentiated analysis of urea and SCr alterations provides important information about renal function. Thereby, a key point is to distinguish between azotemia and developing uremia. Neglecting potential confounders of SCr-/urea-levels considerably influences AKI studies and might lead, at least in part, to the prevailing inconsistency.

In summary, RRT initiation remains an individual clinical decision based on expertise and special patient characteristics, leading to wide variation in practice. Furthermore, it is not RRT timing, but rather the early identification of patients without autonomous renal recovery, which may be the major point of interest. In the future, a set of biomarkers may help us to solve these issues and might elucidate septic and heterogeneous AKI pathophysiology. Until then, it is still a long way down the road. A better understanding of an appropriate use of RRT besides absolute indications is needed to avoid unnecessary RRT for higher patient safety and lower health care costs.

## Figures and Tables

**Table 1:** AKI staging by Kidney Disease Improving Global Outcomes (KDIGO) 2012 [29].

AKI	Stage 1	Stage 2	Stage 3
Serum creatinine	I: 1.5–1.9 times baseline ^a^ II: ≥0.3 mg/dL increase within 48 h	I: 2.0–2.9 times baseline ^a^	I: ≥3 times baseline ^a^ II: Increase ≥4 mg/dL III: Initiation of RRT
Urine output	III: <0.5 mL/kg/h for 6–12 h	II: <0.5 mL/kg/h for ≥12 h	IV: <0.3 mL/kg/h for ≥24 h V: Anuria ≥12 h

AKI, Acute kidney injury; RRT, Renal replacement therapy; ^a^ SCr, Rise within seven days.

**Table 2:** Overview of promising “renal damage” biomarkers [39,40,41,42,43,44].

“Renal Damage” Biomarkers			
“Indicate renal damage at a certain time point and give insights in heterogeneous AKI pathophysiology”			
Molecular Weight	Origin	Kinetics	Evidence
NGAL			
25 kDa	thick ascending limb and collecting ducts	Peak approximately 6 h after injury Detectable 3 h after injury Sustained elevation up to 5 days	Reno-protective and anti-apoptotic Bind to iron-siderophore complexes Bacteriostatic agent Iron-trafficking in developing renal epithelium Induces tubulogenesis in nephrogenesis
KIM-1			
38.7 kDa	Proliferating, dedifferentiated epithelial cells in proximal tubule	Peak 48 h (2–3 days) after injury	Reno-protective Confers kidney cells to phagocytic cell type Promote phagocytosis, role in tubular regeneration and renal recovery Distinguish period of injury and recovery
IL-18			
22 kDa	Predominantly immune cells	Peak 12–18 h after injury First detection within 6 h after injury	Promotes renal injury Promotes inflammation via NF-κB, release of TNFα, iNOS and chemokines
L-FABP			
14 kDa	Predominantly proximal tubule	Peak within 6 h after injury	Reno-protective Antioxidant Gene induction by hypoxia = marker for renal hypoxia protection from reactive oxygen species promotes metabolism of fatty acids
TIMP-2			
24 kDa	Proximal and distal tubule, predominantly distal	Peak probably within 12–24 h after injury	Reno-protective and pro-recovery role Predicts AKI KDIGO stage 2–3 within 12 h Induces G1 cell cycle arrest prevents cell death role in nephrogenesis special role not fully understood
IGFBP-7			
29 kDa	In AKI proximal and distal tubule, predominantly proximal Ubiquitously expressed	Peak probably within 12–24 h after injury	Reno-protective by inducing G_1_-cell cycle arrest Potentially impairs renal perfusion and recovery Predicts AKI KDIGO stage 2–3 within 12 h G_1_-cell cycle arrest marker Known as tumor suppressor and regulator of cellular senescence Potential deleterious effect as IGF-1 receptor antagonist

IFGBP-7, Insulin-like growth factor-binding protein-7; iNOS, Inducible nitric oxide synthase, IL 18, Interleukin 18 kDa; KIM-1, Kidney injury molecule-1; L-FABP, Liver-type fatty acid-binding protein; TIMP-2, Tissue inhibitor of metalloprotease-2; TNFα, Tumor necrosis factor alpha.

**Table 3:** Confounders of creatinine interpretation.

Rise in Creatinine	Fall in Creatinine
Acute rise in creatinine:	Acute fall or attenuated rise in creatinine:
Dietary creatine/meat intake [105] Enhanced creatinine formation ○ Rhabdomyolysis [86,87] Decreased GFR ○ AKI [77] Reduced tubular secretion [88,89,90,91] ○ Trimethoprim, cimetidine, ranitidine, dronedarone [49,92,93,94,95]	Reduced creatinine formation ○ In sepsis [81] ○ Acute liver failure [80] Increased volume of distribution [48,82,83,84,106] ○ Acute fluid overload
**Chronic creatinine elevation:**	**Chronic reduction in creatinine:**
Enhanced creatinine formation [76] ○ Afro-American ethnicity ○ Muscular body habitus Decreased GFR ○ CKD	Low dietary protein intake [79] Reduced creatinine formation/low muscle mass/catabolic metabolism [49,76,78,79] ○ Amputation ○ Female sex, old age ○ Muscle wasting ○ Critical illness, malnutrition ○ Insufficient protein supply ○ Immobilization ○ Liver cirrhosis
**False creatinine elevation:**	**False reduction in creatinine:**
Jaffé assay ○ Hyperglycemia & diabetic ketoacidosis [98,99,100,101] ○ Delayed centrifugation ○ Hemolysis [77] ○ Drugs (cefoxitin, flucytosin, dopamine) [96,97,103] ○ Ig M gammopathy, uric acid [77] Enzymatic assay ○ High total protein, lidocaine [77]	Jaffé assay ○ Hyperbilirubinemia [80,102,104] Enzymatic assay ○ Hyperbilirubinemia [80,102,104]

AKI, Acute kidney injury; CKD, Chronic kidney disease; GFR, Glomerular filtration rate.

**Table 4:** Overview of relevant studies investigating the timing of renal replacement therapy after 1990.

Author	Study Design	Patients Early/Late	Purposed Early Criteria	Purposed Late Criteria	Cutoff before Early RRT	Cutoff before Late RRT	Mortality Early/Late
Gaudry et al., 2016 [74]	RCT	311/308	After randomization + AKI Stage III	Hyperkalemia > 6 mmol/L, metabolic acidosis pH < 7.15, pulmonary edema > 5 L O_2_, BUN > 112 mg/dL, Oliguria >72 h	BUN 52 mg/dL SCr 3.27 mg/dL	BUN 90 mg/dL SCr 5.33 mg/dL	49%/50% (*p* = 0.790)
Zarbock et al., 2016 [126]	RCT	112/119	Within 8 h of KDIGO stage 2 diagnosis	Within 12 h of stage 3 diagnosis	BUN 38.5 mg/dL SCr 1.5 mg/dL	BUN 47.5 mg/dL 2.4 mg/dL	39%/55% (*p* = 0.030)
Wald et al., 2015 [125]	RCT	48/33	Within 12 h after fulfilling study criteria	Potassium > 6 mmol/L, bicarbonate < 10 mmol/L, Horowitz < 200+ infiltrates X-ray	Urea 115.9 mg/dL SCr 3.68 mg/dL	Urea 161.6 mg/dL SCr 4.57 mg/dL	38%/37% (*p* = 0.920)
Jamale et al., 2013 [116]	RCT	102/106	BUN > 70 mg/dL or SCr > 7 mg/dL	Clinically indicated or jugged by nephrologist	BUN > 71.7 mg/dL SCr > 7.4 mg/dL	BUN 100.9 mg/dL SCr 10.4 mg/dL	21%/12% (*p* = 0.200)
Sugahara et al., 2004 [131]	RCT	14/14	3h after UO < 30 mL/h	2 h after UO < 20 mL/h	SCr 2.9 mg/dL	SCr 3.0 mg/dL	Survival 86%/14%, (*p* = 0.010)
Durmaz et al., 2003 [132]	RCT	21/23	10% increase of SCr after surgery	50% increase or UO < 400 mL/24 h	BUN 53.7 mg/dL SCr 3.1 mg/dL	BUN 65.0 mg/dL SCr 4.3 mg/dL	4.7%/ 0% (*p* = 0.048)
Bouman et al., 2002 [133]	RCT	35/36	within 12 h: UO < 30 mL/h and 3 h CrCl < 20 mL/min	Urea > 40 mmol/L or K > 6.5 mmol/L or severe pulmonary edema	Urea 17.1 mmol/L	Urea 37.4 mmol/L	Survival 67%/75% (*p* = 0.800)
Vaara et al., 2014 [123]	Prospective cohort	105/134	RRT without classic indications = pre-emptive	Classic RRT indications	Urea 19.1 mmol/L SCr 2.6 mg/dL	Urea 23.2 mmol/L SCr 3.7 mg/dL	27%/49% (*p* = 0.010)
Leite et al., 2013 [124]	Prospective cohort	64/86	<24 h after AKIN 3	≥24 h after AKIN 3	Urea 100.1 mg/dL SCr 2.7 mg/dL	Urea 108.2 mg/dL SCr 2.8 mg/dL	51%/82% (*p* = 0.002)
Bagshaw et al., 2009 [134]	Prospective cohort	618/619 618/618	Median Urea of all patients Median SCr of all patients		Urea < 24.2 mmol/L SCr < 3.5 mg/dL	Urea >24.2 mmol/L SCr >3.5 mg/dL	62%/59% (*p* < 0.001)
Liu et al., 2006 [135]	Prospective cohort	122/121	BUN ≤ 76 mg/dL	BUN > 76 mg/dL	BUN 47 mg/dL SCr 3.4 mg/dL	BUN 115 mg/dL SCr 4.7 mg/dL	Survival 65%/59% (*p* = 0.090)
Gaudry et al., 2015 [120]	Retrospective cohort	34/27	UO < 100 mL/8 h and no response to 50 mg furosemide	SCr > 5 mg/dL or K >5.5 mEq/L irrespective of UO	NR	NR	24%/56% (*p* = 0.016)
Jun et al., 2014 [121]	Retrospective cohort	I: 109 II: 110 III: 109 IV: 111	Time between meeting Rifle I and Randomization (=CRRT) I: <7.1 h II: ≥7.1 to 17.6 h III: ≥17.6 to 46 h IV: ≥46 h		I: urea 103.3 mg/dL, SCr 2.97 mg/dL (reference group) II: urea 102.7 mg/dL, SCr 2.81 mg/dL III: urea 124.7 mg/dL, SCr 3.59 mg/dL IV: urea 173 mg/dL, SCr 3.75 mg/dL		I: 36% II: 39% III: 37% IV: 40% (*p* = 0.923)
Fernandez et al., 2011 [9]	Retrospective cohort	101/102	Within first 3 days after surgery	After the third day	NR	NR	53%/80% (*p* < 0.001)
Ji et al., 2011 [136]	Retrospective cohort	34/24	Within 12 h UO ≤ 0.5 mg/kg/h after surgery + 50% increase in baseline of crea and urea	12 h after the onset of early criteria	BUN 60.8 mg/dL SCr 2.8 mg/dL	BUN 93.6 mg/dL SCr 4.5 mg/dL	9%/38% (*p* = 0.020)
Carl et al., 2010 [137]	Retrospective cohort	85/62	BUN < 100 mg/dL	BUN ≥ 100 mg/dL	BUN 66 mg/dL SCr 5 mg/dL	BUN 137 mg/dL SCr 5.8 mg/dL	52%/68% (*p* = NR)
Iyem et al., 2009 [138]	Retrospective cohort	95/90	UO ≤ 0.5 mL/kg/h after surgery and 50% increase of baseline crea and urea	48 h after the onset of early criteria	BUN 54.6 mg/dL SCr 2.1 mg/dL	BUN 68.2 mg/dL SCr 2.9 mg/dL	5%/7% (*p* = NR, reported as not significant)
Shiao et al., 2009 [139]	Retrospective cohort	51/47	RIFLE Risk	RIFLE Injury/Failure	BUN 68.8 mg/dL SCr 3.3 mg/dL	BUN 81.9 mg/dL SCr 3.8 mg/dL	43%/75% (*p* = 0.002)
Manche et al., 2008 [140]	Retrospective cohort	56/15	Hyperkaliemia	UO <0.5 mL/kg/h	Urea 14.4 mmol/L SCr 2.64 mg/dL	Urea 35.2 mmol/L SCr 4.56 mg/dL	Survival 75%/13% (*p* < 0.001)
Andrade et al., 2007 [141]	Retrospective cohort	18/15	On admission	24 h	Urea 107 mg/dL	Urea 153 mg/dL	17%/67% (*p* = 0.010)
Wu et al., 2007 [142]	Retrospective cohort	54/26	BUN < 80 mg/dL	BUN > 80 mg/dL	BUN 46.2 mg/dL SCr 2.9 mg/dL	BUN 118.8 mg/dL Scr 4.7 mg/dL	63%/85% (*p* = 0.040)
Piccinni et al., 2005 [143]	Retrospective cohort	40/40	Within 12 h after admission and diagnosis of septic shock	Classic RRT indications	BUN 120 mg/dL SCr 1.8 mg/dL	BUN 110 mg/dL SCr 1.7 mg/dL	Survival 55%/27% (*p* = 0.005)
Demirkilic et al., 2004 [144]	Retrospective cohort	27/34	UO < 100 mL/8 h despite 50 mg furosemide	SCr > 5 mg/dL or K > 5.5 mmol/L	NR	NR	24%/56% (*p* = 0.016)
Elahi et al., 2004 [145]	Retrospective cohort	28/36	UO < 100 mL/8 h despite furosemide	Urea ≥ 30 mmol/L, Crea ≥ 250 mmol/L or K ≥ 6.5 mmol/L	Urea 23.9 mg/dL SCr 3.7 mg/dL	Urea 26.8 mg/dL SCr 4.3 mg/dL	43%/22% (*p* = < 0.050)
Gettings et al. 1999 [146]	Retrospective cohort	51/49	BUN < 60 mg/dL	BUN ≥ 60 mg/dL	BUN 43 mg/dL SCr 2.69 mg/dL SCr 5.73 mg/dL ^a^	BUN 94 mg/dL SCr 3.59 mg/dL SCr 6.5mg/dL ^a^	Survival 39%/20% (*p* = 0.041)

BUN, Blood urea nitrogen; d, Days; NR, Not reported; *p*-value, ≤0.05 statistical significance; RRT, Renal replacement therapy; RCT, Randomized controlled trial; UO, Urine output. ^a^ Patients with rhabdomyolysis.

**Table 5:** Non-dialytic management of acute kidney injury.

Strategy	Therapeutic Measures
Early AKI recognition [148,149,150]	Remove obstruction in post-renal AKI
	Appropriate fluid removal in pre-renal AKI
	Avoidance of contrast agents and nephrotoxins
Enhanced AKI monitoring [148,149,150]	Monitoring renal function parameter, electrolytes and urine output
Avoidance of nephrotoxines [148,149,150]	Check medication
	Discontinue nephrotoxic drugs
Appropriate intravenous fluid administration [151,152]	Avoid sodium- and chloride-rich solution
	Avoid hydroxyethyl starches
	Use balanced electrolyte solutions
Preventing fluid overload/hyperkaliemia [75,151,153,154]	Appropriate use of diuretics
	Use of cation exchanger and loop diuretics
	Cautious fluid administration
	Low salt diet
Limiting potassium and phosphate intake	Low potassium and phosphate diet
	Appropriate parenteral nutrition
	Avoid potassium and phosphate-rich intravenous solutions or oral supplements
Optimization of renal perfusion pressure [155,156,157]	Mean arterial pressure ≥ 65 or ≥ 80–85 mmHg in patients with chronic hypertension
	Fluid administration
	Use of catecholamines
Recognize and avoid azotemia	Check for BUN/urea to SCr dissociation
	Exclude low SCr production (muscle wasting, etc.)
	Avoid excessive, nutritional protein supply
	Evaluate metabolic status (catabolic vs. anabolic)
	Check for gastrointestinal bleeding or steroid therapy

AKI, Acute kidney injury; BUN, Blood urea nitrogen.

## Abbreviations

AKI	Acute kidney injury
AKIN	Acute Kidney Injury Network
BUN	Blood urea nitrogen
CKD	Chronic kidney disease
CKD-EPI	Chronic Kidney Disease Epidemiology Collaboration
COX-1	Cyclooxygenase-1
CrCl	Creatinine clearance
^51^Cr-EDTA	Chromium-ethylenediaminetetraacetic acid
ESRD	End-stage renal disease
GFR	Glomerular filtration rate
eGFR	Estimated glomerular filtration rate
FO	Fluid overload
ICAM-1	Intracellular adhesion molecule-1
ICU	Intensive Care Unit
IGFBP-7	Insulin like growth factor binding protein-7
KDIGO	Kidney Disease Improving Global Outcomes
IL	Interleukin
MDRD	Modification of diet in renal disease
OAT	Organic anion transporter
PMMA	Polymethylmethacrylate
RBF	Renal blood flow
RRT	Renal replacement therapy
SCr	Serum creatinine
TIMP-2	Tissue inhibitor of metalloproteinase-2
TGF	Tubular glomerular feedback
TNFα	Tumor necrosis factor alpha
UO	Urine output
VOD	Volume of distribution
